# Aging and the Prevalence of Polypharmacy and Hyper-Polypharmacy Among Older Adults in South Korea: A National Retrospective Study During 2010–2019

**DOI:** 10.3389/fphar.2022.866318

**Published:** 2022-05-09

**Authors:** Ho Jin Cho, Jungmi Chae, Sang-Heon Yoon, Dong-Sook Kim

**Affiliations:** Health Insurance Review and Assessment Service, Wonju, South Korea

**Keywords:** polypharmacy, hyper-polypharmacy, elder patients, outpatient care, aging

## Abstract

**Background:** Polypharmacy has become a global health problem and is associated with adverse health outcomes in the elderly. This study evaluated the prevalence of polypharmacy and hyper-polypharmacy in elderly patients in South Korea during 2010–2019.

**Methods:** We analyzed the outpatient care of persons aged ≥65 years covered by National Health Insurance (NHI) using NHI claims data from 2010 to 2019. Polypharmacy was defined as the use of ≥5 medications, and hyper-polypharmacy was defined as the use of ≥10 medications, and we examined them over periods of ≥90 days and ≥180 days. The average annual percent change (AAPC) was calculated using Joinpoint statistical software.

**Results:** The prevalence of polypharmacy among ≥90 days of medication use elderly decreased from 42.5% in 2010 to 41.8% in 2019, and the prevalence of hyper-polypharmacy for ≥90 days increased from 10.4% to 14.4%. The prevalence of polypharmacy for ≥180 days increased from 37.8% in 2010 to 38.1% in 2019, and the prevalence of hyper-polypharmacy for ≥180 days increased from 6.4% to 9.4%. The prevalence of polypharmacy for ≥90 days and ≥180 days steadily increased among elderly patients, with AAPCs of 3.7 and 4.5, respectively.

**Conclusion:** The prevalence of polypharmacy for ≥90 days and ≥180 days remained stably high, with rates of about 42 and 38%, respectively, and hyper-polypharmacy increased over the past 10 years in South Korea. Therefore, strategies to address polypharmacy need to be implemented. Further research is also required to identify the clinical outcomes (including mortality risks) associated with polypharmacy.

## Introduction

South Korea is the most rapidly aging country of the Organization for Economic Co-operation and Development (OECD) (OECD, 2021). As of 2020, 15.7% of the population consists of seniors aged 65 years or older, and the proportion is expected to increase to 43.9% by 2060 (Statistics Korea, 2020). Compared to other age groups, older adults more often have multimorbidity and more frequently use medical services (Barnett et al., 2012). Thus, they are more likely to routinely visit multiple medical institutions simultaneously and are thus more vulnerable to drug-related problems such as polypharmacy, the use of inappropriate medications, and adverse drug reactions (Barnett et al., 2012).

Polypharmacy refers to the concurrent use of multiple medications. Despite the lack of a clear universal definition, polypharmacy is often defined as the routine use of 5 or more medications according to the World Health Organization (WHO, 2019). A recent systematic review on definitions of polypharmacy showed that 46.4% of studies used a numerical definition, such as 5 or more medications or 10 or more medications (Masnoon et al., 2017). However, this is a relatively simple definition of polypharmacy, and using only the number of medications could make it difficult to evaluate appropriate polypharmacy (rational prescribing of multiple drugs based on the best available evidence and considering the individual patient context) (Masnoon et al., 2017). Other studies have defined polypharmacy as the prescription of at least 1 medication that is clinically inappropriate or offers no additional benefit (Colley and Lucas, 1993; Carlson, 1996; Chumney and Robinson, 2006). In that sense, it is also necessary to consider the possibility of drug overdoses, prescriptions for drugs that are not necessary, and the duration of mediation use (Colley and Lucas 1993; Carlson 1996; Chumney and Robinson 2006). Several systematic reviews reported that the adverse health outcomes of polypharmacy in older people include a decrease in patient compliance and an increased likelihood of drug-drug interactions and adverse drug reactions, which thereby increase the risk of hospitalization, additional medical expenses and death (Fried et al., 2014; O'Dwyer et al., 2016; Leelakanok et al., 2017; Ming and Zecevic, 2018; Al-Musawe et al., 2019; Katsimpris et al., 2019; Leelakanok and D'Cunha, 2019; Palmer et al., 2019; Davies et al., 2020).

Previous studies in various countries have shown that the prevalence of polypharmacy varied according to the healthcare service setting and definition. The prevalence of excessive polypharmacy or hyper-polypharmacy (10 or more medications) among the elderly was 5.8% in Scotland (Guthrie et al., 2015), 5.4% in Taiwan (Wang et al., 2018), 5.1% in Sweden (Zhang et al.,. 2020), and 1.3% in New Zealand (Nishtala and Salahudeen, 2015). According to previous literature, the prevalence of patients who had been simultaneously prescribed 5 or more medications ranged from 26 to 44% (Khezrian et al., 2020). The prevalence of polypharmacy (5 or more medications) among the elderly was 20.8% in Scotland (Guthrie et al., 2015), 19.1% for chronic polypharmacy within consecutive 6 months in Poland (Kardas et al., 2021), 41.2% in Switzerland (Blozik et al., 2018), 36.8% in the United States (Young et al., 2021), 28.7% in Japan (Mabuchi et al., 2020), and 19% in Sweden (Zhang et al., 2020).

In studies that reviewed the status of polypharmacy among seniors in South Korea, Kim et al., 2014 and Nam et al., 2016 reported that 86.4 and 65.2% of seniors had been prescribed 6 or more simultaneous drugs at least once in a single year (Kim et al., 2014; Nam et al., 2016), and Park et al., 2016 and Chang et al., 2020 reported that 44.1 and 46.6% of South Koreans aged 65 years or over were prescribed 5 or more medications (Park et al., 2016; Chang et al., 2020).

However, no studies have analyzed trends in polypharmacy by year across the entire population. Therefore, we aimed to analyze yearly trends in polypharmacy from 2010 to 2019 for the entire elderly population of South Korea. Furthermore, we distinguished between polypharmacy and hyper-polypharmacy for the analysis, based on both the number of medications taken and the duration of use.

## Methods

### Data Source

We conducted a retrospective cohort study of the elderly population using the National Health Insurance (NHI) data.

The NHI data was the details for claims from medical institutions and then the Health Insurance Review & Assessment Service (HIRA) reviews these for payment. In Korea, the NHI covers 97% of the Korean population, and the claims data include patients’ demographic characteristics, as well as the diagnosis codes of diseases, the international non-proprietary names of drugs, the prescribed doses per day, and the days of therapies.

In the analysis, we used outpatient prescriptions from January 2010 to December 2019 from medical institutions including tertiary hospitals, secondary hospitals, general hospitals, nursing homes, clinics, and public health centers. In the case of people who had been admitted to hospitals, we included their outpatient prescriptions. The proportion of subject elderly patients was 95% of the total elderly population (total elderly population was 5,429,802 in 2010 and 8,018,762 in 2019).

### Patient Population and Definition of Polypharmacy

The study population included all individuals aged 65 years or older, and all outpatient medical encounters of this population were used in the analysis. This study analyzed orally administered drugs that had been listed in the national health insurance benefits scheme between 1 January 2010, and 31 December 2019.

The outcome measures of this study were the number of patients with polypharmacy patients and the number of patients with hyper-polypharmacy patients according to their use of medication for ≥90 days and ≥180 days from 2010 to 2019. We differentiated between polypharmacy (being prescribed ≥5 or more drugs) and hyper-polypharmacy (being prescribed ≥10 drugs).

The number of active substances prescribed each day was determined for each patient unit over a period of 365 days. We calculated the number of medications with the same active substances regardless of dose or formulation, such as a tablet or sustained-release tablet. We classified a patient as experiencing continuous polypharmacy or hyper-polypharmacy if the total number of days of the patient’s multiple drug use (5 or more, 10 or more) was accumulative 90 or 180 in the same year. Thus, if a patient used 6 drugs for 50 days cumulatively in year t and 6 drugs for 95 days in year t+1, he or she would be classified into the non-polypharmacy group in year t and the continuous polypharmacy group in year t+1. In addition, if the treatment started in October and the prescription continued until March of the following year, the 3-months prescription in year t and the 3-months prescription in t+1 would be attributed to each separate year.

### Statistical Analysis

A descriptive statistical analysis was performed. Differences in polypharmacy and hyper-polypharmacy across sex, age groups, and chronic diseases were examined between 2010 and 2019. In addition, to examine changes in the prevalence of polypharmacy and hyper-polypharmacy by year, Joinpoint regression analysis was performed. We calculated the annual percent change (APC) and average annual percent change (AAPC) using the proportion of people with polypharmacy to assess the temporal trends of polypharmacy and hyper-polypharmacy. The APC is a way to characterize trends in prevalence over time. To estimate the APC for a series of data, regression in a logarithmic scale model is used. The joinpoint model uses statistical criteria to determine when and how often the APC changes. Finding the joinpoint model that best fits the data allows us to determine how long the APC remained constant, and when it changed. The AAPC is a summary measure over a fixed pre-specified interval that makes it possible to use a single number to describe the average APCs over a period of multiple years (NIH).

The annual percent change (APC) was obtained for each trend line using the Joinpoint program developed by the National Cancer Institute of the United States, and the average APC (AAPC) value was obtained to assess the trend by year (Clegg et al., 2009). A *p*-value of <0.05 was considered to indicate statistical significance.

## Results

### General Characteristics of Elderly Patients by Year

As shown in Table 1, from 2010 to 2019, the number of patients aged 65 years or older increased from 5.2 million in 2010 to 7.7 million in 2019. The proportion of patients aged 65–69 years was the highest, with 31.6% (2.4 million) in 2019, and the population aged 85 years or older more than doubled from 0.3 million in 2010 to 0.7 million in 2019, showing the largest increase. There were more women than men, and the number of patients aged 85 years or older gradually increased, reflecting the aging of South Korea’s population. Although the proportion of women among the elderly was higher (56.9% in 2019), the annual increase rate was 6.49% for men, which was higher than that of 4.64% for women.

**TABLE 1:** General characteristics of elderly patients with outpatient prescriptions.

Year	2010		2011	2012	2013	2014	2015	2016	2017	2018	2019		Annual Increase Rate (%)
No. of patients	5,201,276		5,411,592	5,713,734	5,974,108	6,235,700	6,474,327	6,693,802	7,046,476	7,357,078	7,722,213		5.39
Sex													
Male	2,099,908	(40.4)	2,201,909	2,345,343	2,473,240	2,599,708	2,717,929	2,825,863	2,997,317	3,148,275	3,326,394	(43.1)	6.49
Female	3,101,368	(59.6)	3,209,683	3,368,391	3,500,868	3,635,992	3,756,398	3,867,939	4,049,159	4,208,803	4,395,819	(56.9)	4.64
Age													
65–69 years	1,800,317	(34.6)	1,789,202	1,799,905	1,882,353	1,972,321	2,079,577	2,132,322	2,239,501	2,306,639	2,440,720	(31.6)	3.95
70–74 years	1,498,662	(28.8)	1,570,977	1,707,598	1,735,553	1,734,561	1,722,208	1,715,513	1,727,090	1,814,482	1,904,724	(24.7)	3.01
75–79 years	1,008,529	(19.4)	1,090,106	1,160,533	1,232,649	1,306,653	1,344,631	1,413,640	1,537,649	1,573,012	1,578,401	(20.4)	6.28
80–84 years	553,756	(10.6)	593,460	642,702	685,867	743,492	811,064	878,909	937,345	1,003,923	1,072,901	(13.9)	10.42
≥85 years	340,012	(6.5)	367,847	402,996	437,686	478,673	516,847	553,418	604,891	659,022	725,467	(9.4)	12.60
Mean ± SD	73.3 ± 6.4		73.5 ± 6.4	73.6 ± 6.5	73.7 ± 6.6	73.8 ± 6.7	73.9 ± 6.7	74.1 ± 6.8	74.1 ± 6.9	74.2 ± 7.0	74.2 ± 7.1			
Disease														
Hypertension	2,581,068	(49.6)	2,698,944	2,871,014	3,020,143	3,127,229	3,251,965	3,377,110	3,520,861	3,690,716	3,880,467	(50.3)	5.59	
Cardio-cerebrovascular diseases	708,464	(13.6)	738,118	769,820	794,857	808,563	824,746	842,376	865,736	908,699	965,318	(12.5)	4.03	
Hyperlipidemia	861,160	(16.6)	1,005,204	1,143,690	1,286,479	1,455,729	1,623,152	1,839,357	2,019,675	2,223,874	2,464,689	(31.9)	20.69	
Diabetes mellitus	1,101,025	(21.2)	1,199,610	1,296,502	1,390,443	1,471,894	1,555,136	1,646,875	1,757,277	1,873,910	2,011,237	(26.0)	9.19	
Gastric ulcer/gastroesophageal reflux disease	900,849	(17.3)	921,419	972,808	955,284	938,610	926,121	924,474	919,140	926,743	901,305	(11.7)	0.01	
Chronic renal failure	53,226	(1.0)	66,545	81,899	93,443	101,129	113,049	129,431	144,899	165,262	187,896	(2.4)	28.11	
Liver disease	404,017	(7.8)	429,352	467,627	500,677	511,073	542,900	585,088	622,770	706,072	804,115	(10.4)	11.00	
Respiratory disease	772,877	(14.9)	813,996	924,936	904,180	939,409	957,361	944,269	934,592	1,000,483	978,788	(12.7)	2.96	
Cancer	3,387,661	(7.5)	366,883	407,504	446,303	480,045	515,727	555,593	599,626	652,336	705,116	(9.1)	9.10	
Musculoskeletal disease	2,078,698	(40.0)	2,194,343	2,329,917	2,442,143	2,550,716	2,652,127	2,771,548	2,904,274	3,054,021	3,251,092	(42.1)	6.27	
Dementia	262,711	(5.1)	312,570	370,475	429,201	482,337	542,291	622,885	721,893	833,164	946,865	(12.3)	28.94	
Fracture	361,346	(6.9)	362,549	401,783	447,012	450,389	471,148	488,964	528,840	568,701	600,456	(7.8)	7.35	
Healthcare utilization														
Healthcare spending (billion USD)	3.2		3.5	3.8	4.2	4.6	4.9	5.4	6.0	6.9	7.9		16.33	
Prescription spending (billion USD)	2.9		3.1	3.1	3.2	3.4	3.7	4.1	4.5	4.9	5.5		9.73	
Number of visits (day)	28.4		28.9	31.0	31.0	30.9	30.3	30.4	30.0	30.1	30.3		0.74	
Number of medications per day, Mean ± SD (Median)	4.7 ± 2.4 (4.2)		4.6 ± 2.4 (4.1)	4.7 ± 2.5 (4.1)	4.7 ± 2.6 (4.1)	4.7 ± 2.6 (4.2)	4.7 ± 2.6 (4.2)	4.8 ± 2.7 (4.2)	4.8 ± 2.7 (4.2)	4.9 ± 2.8 (4.3)	4.9 ± 2.9 (4.3)		0.47	
Spending per patient (USD)	618.3		645.8	671.7	700	733.3	753.3	800.8	850	936.7	1,029.2		7.38	
Prescription spending per patient (USD)	561.7		579.2	535.0	535.8	549.2	565.8	612.5	635	672.5	710		2.93	

SD: standard deviation.

The number of patients with hypertension was the highest (3.8 million in 2019), followed by musculoskeletal diseases (3.2 million in 2019). The proportion of patients with dementia showed the largest increase from 5.1% in 2010 to 12.3% in 2019, and the annual rate of increase was the highest (28.94%), followed by chronic renal failure, the prevalence of which increased from 1.0% in 2010 to 2.4% in 2019, with an annual increase rate of 28.11%, and hyperlipidemia (the prevalence of which increased significantly from 16.6% in 2010 to 31.9% in 2019, with an annual increase rate of 20.69%). Meanwhile, the percentage of patients with gastric ulcers/gastroesophageal reflux disease decreased from 17.3 to 11.7%.

An overall increasing trend was found in the number of outpatient care visits, outpatient care spending, prescription days, and prescription spending of patients aged 65 years or older. The number of outpatient visits increased from 28.4 days in 2010 to 31 days in 2013 and then slightly decreased. The outpatient care spending per patient steadily increased, while the number of prescription medications per day declined in 2011 and increased slightly from 4.7 in 2010 to 4.9 in 2019. The outpatient care spending per patient increased during 2010–2019, whereas the prescription spending per patient decreased between 2012 and 2014 and thereafter increased.

### Annual Prevalence of Polypharmacy in Elderly Patients

As shown in Table 2, the prevalence was approximately 41.8% for ≥90-days polypharmacy and approximately 14.4% for ≥90-days hyper-polypharmacy in 2019. The prevalence was approximately 38.1% for ≥180-days polypharmacy and approximately 9.4% for ≥180-days hyper-polypharmacy.

**TABLE 2:** The trend of the prevalence of polypharmacy and hyper-polypharmacy among the elderly patients prescribed ≥90 and ≥180 days in 2010–2019 period.

Year	2010	2011	2012	2013	2014	2015	2016	2017	2018	2019	AAPC (95% CI)
Patients aged ≥65 years	5,201,276	5,411,592	5,713,734	5,974,108	6,235,700	6,474,327	6,693,802	7,046,476	7,357,078	7,722,213	3.6 (3.4, 3.8)
Elderly prescribed ≥90 days											
Patients aged ≥65 years prescribed ≥90 days	3,866,631	4,129,037	4,461,220	4,746,381	4,996,503	5,224,343	5,487,779	5,847,509	6,171,951	6,570,508	
Polypharmacy (%)	1,645,208	1,753,525	1,886,321	1,999,604	2,097,061	2,181,402	2,296,197	2,436,875	2,584,820	2,748,452	−0.2 (−0.3, −0.1)
	(42.5)	(42.5)	(42.3)	(42.1)	(42.0)	(41.8)	(41.8)	(41.7)	(41.9)	(41.8)	
Hyper-polypharmacy (%)	403,249	435,327	491,481	547,690	589,920	629,325	689,583	763,321	846,462	944,458	3.7 (3.2, 4.2)
	(10.4)	(10.5)	(11.0)	(11.5)	(11.8)	(12.0)	(12.6)	(13.1)	(13.7)	(14.4)	
Elderly prescribed ≥180 days											
Patients aged ≥65 years prescribed ≥180 days	3,322,548	3,584,969	3,924,215	4,216,244	4,459,449	4,676,878	4,947,263	5,305,607	5,630,649	6,031,756	
Polypharmacy (%)	1,256,349	1,353,377	1,475,772	1,587,584	1,671,917	1,749,234	1,863,607	1,997,042	2,135,168	2,296,323	0.1 (−0.0, 0.2)
	(37.8)	(37.8)	(37.6)	(37.7)	(37.5)	(37.4)	(37.7)	(37.6)	(37.9)	(38.1)	
Hyper-polypharmacy (%)	212,106	230,202	267,883	305,300	331,840	357,176	396,994	445,685	501,117	567,651	4.5 (4.1, 4.9)
	(6.4)	(6.4)	(6.8)	(7.2)	(7.4)	(7.6)	(8.0)	(8.4)	(8.9)	(9.4)	

AAPC: average annual percent change, CI, confidence interval.

**FIGURE 1:**
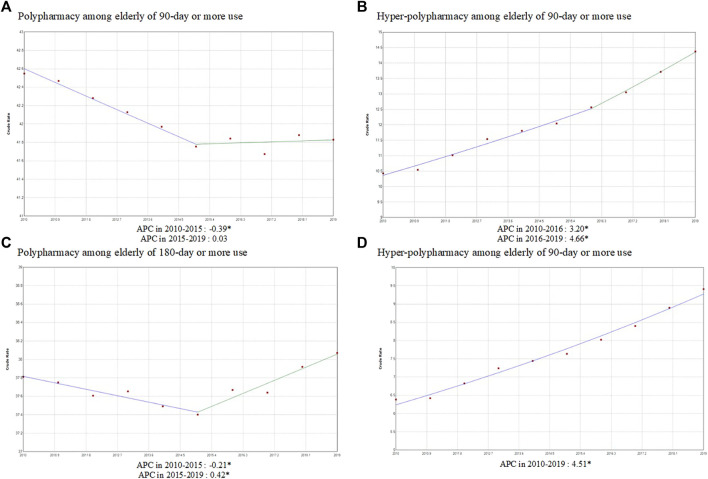
The trend of the prevalence of polypharmacy and hyper-polypharmacy among the elderly prescribed ≥90 days and ≥180 days from 2010 to 2019. APC: Annual percent change, CI, confidence interval.

Figure 1 shows the AAPCs in the trends of the prevalence of polypharmacy and hyper-polypharmacy. Among the elderly with prescriptions for over 90 days, there was a significantly decreasing trend in polypharmacy from 2010 to 2015. But there were no notable changes after 2015, and the AAPC for 2010 to 2019 was −0.2, indicating a decreasing trend in polypharmacy. Meanwhile, the rate of hyper-polypharmacy continued to increase, with an AAPC of 3.7. Specifically, the rate of hyper-polypharmacy increased more rapidly from 2016 to 2019 than it did from 2010 to 2016.

In case of the 180-days polypharmacy, there was a significantly decreasing trend in polypharmacy from 2010 to 2015, but there was an increasing trend after 2015. The AAPC for 2010 to 2019 was 0.1, but it was not significant given the large change in the trend. In contrast, hyper-polypharmacy continued to increase, with an AAPC of 4.5.

### The Prevalence of Polypharmacy by Subgroup

**FIGURE 2:**
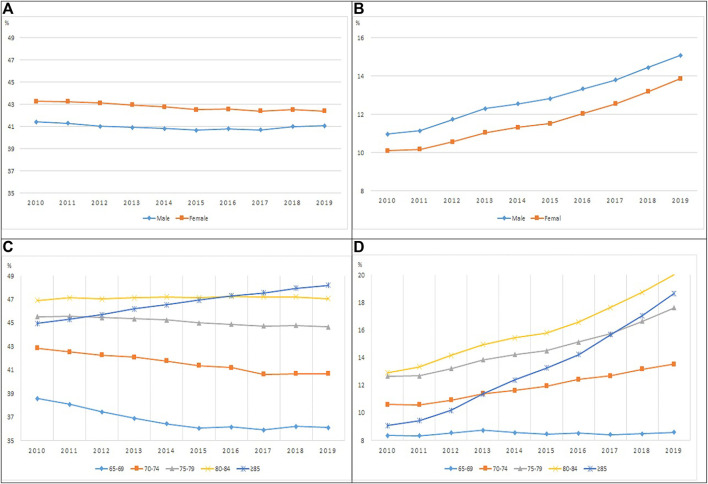
The proportion of polypharmacy and hyper-polypharmacy among ≥90 days use elderly by sex and age group **(A, C)** Polypharmacy, **(B, D)** Hyper-polypharmacy).

**FIGURE 3:**
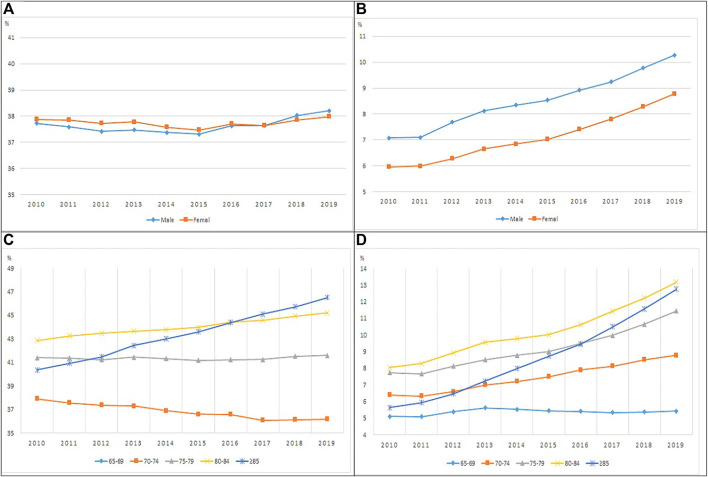
The proportion of polypharmacy and hyper-polypharmacy among ≥180 days use elderly by sex and age group **(A, C)**: Polypharmacy, **(B, D)**: Hyper-polypharmacy).

Figures 2, 3 show the proportions of polypharmacy and hyper-polypharmacy for ≥90 days and ≥180 days among older adults with medication use. Polypharmacy for ≥90 days decreased among both men and women, while ≥180-days polypharmacy slightly increased in men. Both ≥90-days and ≥180-days hyper-polypharmacy increased in men and women. Among the elderly who received prescriptions more than 90 days and for more than 180 days, polypharmacy (use of 5 or more medications) gradually decreased in other age groups, but increased in those aged 80–84 years, and showed a sharp increase in those aged 85 years and older. Hyper-polypharmacy (use of 10 or more medications) decreased among those aged 65–69 years, increased sharply in those aged 70 years and older, and nearly doubled in those aged 85 years and older. In addition, hyper-polypharmacy sharply increased among those aged 85 years or older.

## Discussion

To our knowledge, this is the first population-level study conducted in South Korea targeting the entire elderly population that received prescriptions in the outpatient settings to investigate the 10-years trend of polypharmacy and hyper-polypharmacy among the elderly. Notably, in our study, we considered the number of medications prescribed and the duration for which the medications were taken when defining polypharmacy.

In our study, the prevalence of polypharmacy and hyper-polypharmacy in the elderly was 41.8 and 14.4%, respectively, for ≥90 days, and 38.1 and 9.4%, respectively, for ≥180 days. These results for polypharmacy are similar or higher to those of previous studies, which found that 26–44% of the elderly patients had taken 5 or more medications (Payne et al., 2014; Guthrie et al., 2015; Page et al., 2019; Khezrian et al.. 2020; Mabuchi et al., 2020; Zhang et al., 2020; Kardas et al., 2021; Kardas et al., 2021; Young et al., 2021) and the polypharmacy prevalence in community-dwelling older adults ranged from 7 to 45% (Hsu et al., 2021). The prevalence of hyper-polypharmacy was higher than those reported by other studies (Guthrie et al., 2015; Nishtala and Salahudeen. 2015; Wang et al., 2018; Zhang et al., 2020). However, previous studies conducted in South Korea reported a wide range (44.1–86.4%) of the proportion of individuals aged 65 years or over who were prescribed 5 or more medications (Kim et al., 2014; Nam et al., 2016; Park et al., 2016; Chang et al., 2020). The difference may be because the current study considered both the days of therapy and the number of medications simultaneously, whereas two other studies defined polypharmacy as being prescribed 6 or more drugs at least once in a single year (Kim et al., 2014; Nam et al., 2016).

Second, among the elderly who received prescriptions for more than 90 or 180 days, polypharmacy and hyper-polypharmacy showed a sharp increase in those aged 85 years and older. Due to population aging, the use of multiple drugs in those aged 85 years or older is showing a rapid increase. Polypharmacy fluctuated by year, but hyper-polypharmacy continued to increase. Among the elderly who had prescriptions for more than 180 days, the proportion of men with polypharmacy increased after 2016 compared to women. Since 2016, the NHI benefit scheme has been expanded for four diseases (cardiovascular and cerebrovascular diseases, cancer, and rare diseases) and the number of prescription medicines increased because medicines that were previously not covered were included under NHI benefits.

Third, the previous studies reported that age, number of drugs at admission, hypertension, ischemic heart disease, heart failure, and chronic obstructive pulmonary disease were independently associated with polypharmacy (Nobili et al., 2011). In the current study, most chronic diseases increased yearly except gastric ulcer/gastroesophageal reflux disease. Through this study, we present empirical evidence that the increase in life expectancy has led to an increase in the use of multiple drugs. The medication safety is an important factor that must be considered when treating the older population, particularly those vulnerable to polypharmacy (Avery et al., 2012), and integrated patient-centered management should be implemented.

In addition, we found that the number of outpatient visits and the outpatient care spending per patient steadily increased, whereas the prescription spending per patient decreased between 2012 and 2014 and then increased thereafter. The temporary reduction of prescription spending per patient during 2012–2014 occurred due to the drug price regulation policy implemented from April 2012 to December 2014 which decrease the price of the already listed generic medicines.

To the best of our knowledge, the current study investigated the overall annual changes in polypharmacy and hyper-polypharmacy considering the duration of therapy among the elderly population in South Korea using NHIS claims data. The NHIS reimburses medical institutions on a fee-for-service basis, and the average number of doctor consultations per person was 17.2 visits in 2019, which is the highest rate of medical service usage among all other OECD countries (with an average of 6.6 visits across 33 countries) (OECD 2021).

Also due to the high access to NHI and high healthcare utilization in the Korean medical settings, even mild diseases such as the common cold are treated at medical institutions in South Korea, therefore it is a strength that most drugs prescribed to patients were likely included in the analysis. Furthermore, in this study, all injuries and diseases claimed per patient visit were collected for each patient to identify the presence or absence of injuries and diseases in the subjects. Since all major and minor injuries and diseases experienced by patients over a period of 1 year were included, it can be assumed that all chronic diseases experienced by patients were included. In addition, we attempted to analyze polypharmacy using multiple criteria by conducting a subgroup analysis according to the duration for which medications were taken (≥90 and ≥180 days), and the average number of medications patients took on a daily basis (5 or more and 10 or more medications) during a 1-year period.

Nevertheless, this study has the following limitations. First, since only outpatient injuries and diseases and outpatient prescription records were analyzed, longer-term hospitalization were not included in the analysis. Therefore, the prevalence of polypharmacy was calculated by summing the number of outpatient prescription drugs, excluding drugs administered in an inpatient setting. Since the current study only included the number of outpatient prescriptions, including the number of drugs received during hospitalization would cause the number of drugs actually taken to increase. Second, the analysis was based only on claims data; therefore, we cannot rule out the possibility that some patients did not take the medications prescribed to them. This is likely due to the unique fee-for-service system of South Korea, in which each medication is prescribed by a separate doctor, thereby resulting in frequent overlap in prescriptions for gastrointestinal protective agents and anti-inflammatory drugs. Third, in the current study, polypharmacy was defined based on a numerical definition. Although polypharmacy can be appropriate, the fact that we could not distinguish between appropriate and inappropriate polypharmacy is a limitation of this study. Lastly, since injections are used for a short time and the dose of topical treatments is not high, this study was limited to oral drugs, as in previous studies (Fincke et al., 2005; Chang et al., 2020). Moreover, polypharmacy represents a less-than-desirable state with duplicative medications, drug-to-drug interactions, and inadequate attention to pharmacokinetic and pharmacodynamic principles (Monane, Monane et al., 1997). Thus, complications due to polypharmacy include increased adverse drug reactions and noncompliance (Colley and Lucas 1993).

In conclusion, we investigated annual changes in polypharmacy and hyper-polypharmacy over a period of 10 years using population-level health insurance claims data. As demonstrated in our study, the magnitude of hyper-polypharmacy continued to increase over time, while the prevalence of polypharmacy maintained high rates of about 40%; therefore, it is necessary to establish policy strategies to address polypharmacy. Further studies are also required to identify the clinical outcomes (including mortality risks) associated with polypharmacy.

## Data Availability

The datasets presented in this article are not readily available because Data are not accessed because only those with restrictions can perform the analysis in our institution. Requests to access the datasets should be directed to https://opendata.hira.or.kr/home.do.
