# Supplementary figures and images for: Aging and the Prevalence of Polypharmacy and Hyper-Polypharmacy Among Older Adults in South Korea: A National Retrospective Study During 2010–2019
Source: Front Pharmacol. 2022 May 9;13:866318. doi: 10.3389/fphar.2022.866318 (PMC9124766; doi:10.3389/fphar.2022.866318)

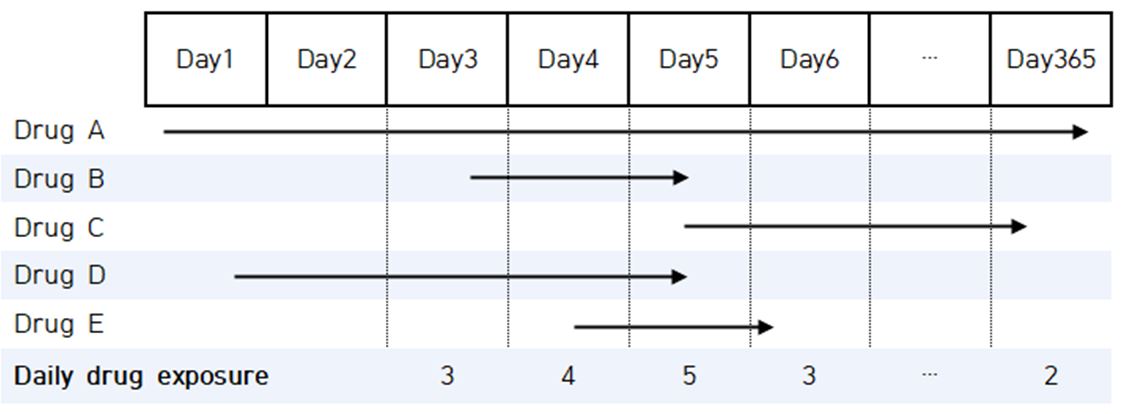

Supplement: Supplementary file 1 [file Image1.JPEG]
